# Antifungal Activity of Linear and Disulfide-Cyclized Ultrashort Cationic Lipopeptides Alone and in Combination with Fluconazole against Vulvovaginal *Candida* spp.

**DOI:** 10.3390/pharmaceutics13101589

**Published:** 2021-09-30

**Authors:** Paulina Czechowicz, Damian Neubauer, Joanna Nowicka, Wojciech Kamysz, Grażyna Gościniak

**Affiliations:** 1 Department of Microbiology, Faculty of Medicine, Wrocław Medical University, 51-368 Wrocław, Poland; joanna.nowicka@umed.wroc.pl (J.N.); grazyna.gosciniak@umed.wroc.pl (G.G.); 2 Department of Inorganic Chemistry, Faculty of Pharmacy, Medical University of Gdańsk, 80-210 Gdańsk, Poland; damian.neubauer@gumed.edu.pl (D.N.); wojciech.kamysz@gumed.edu.pl (W.K.)

**Keywords:** *Candida*, biofilm, Vulvovaginal candidiasis, synergy, lipopeptides, cationic lipopeptides, fluconazole

## Abstract

Vulvovaginal candidiasis (VVC) occurs in over 75% of women at least once during their lifetime and is an infection that significantly affects their health. *Candida* strains resistant to standard azole antifungal therapy and relapses of VVC are more and more common. Hypothetically, biofilm is one of the main reasons of relapses and failure of the therapy. Ultrashort cationic lipopeptides (USCLs) exhibit high antimicrobial activities. Our previous study on USCLs revealed that disulfide cyclization can result in selective antifungal compounds. Therefore, four USCL were selected and their antifungal activity were studied on 62 clinical strains isolated from VVC. The results confirmed previous premises that cyclic analogs have increased selectivity between fungal cells and keratinocytes and improved anticandidal activity compared to their linear analogs against both planktonic and biofilm cultures. On the other hand, linear lipopeptides in combination with fluconazole showed a synergistic effect. It was found that the minimum inhibitory concentrations of the tested compounds in combination with fluconazole were at least four times lower than when used separately. Our results indicate that combination therapy of VVC with USCLs and fluconazole at low non-toxic concentrations can be beneficial owing to the synergistic effect. However, further in vivo studies are needed to confirm this hypothesis.

## 1. Introduction

Vulvovaginal candidiasis (VVC) is the second most common type of vaginal infection, significantly reducing the quality and comfort of women’s lives. According to estimates, more than 75% of women in childbearing age worldwide will experience a symptomatic episode of VVC at least once during their lifetime. Conventional treatments often result in therapeutic failure and/or recurrence of the infection [1,2,3,4,5,6]. Typical recurrent VVC (RVVC), defined as four or more episodes per year, affects at least 10% of patients. At the same time, *Candida* spp. can colonize the vagina and about 1/5 of women are asymptomatic carriers [1,2,4,5]. Generally this fungi are widespread commensals that can be part of the microbiota of mucous membranes and skin where they can cause opportunistic infections, especially in immunocompromised individuals [7]. In the case of VVC, in addition to states of compromised immunity, the most common risk factors are pregnancy, hormone replacement therapy, diabetes, antibiotic therapy, and steroid therapy [1,2]. Meanwhile, the detailed pathomechanism of vaginal mucosa invasion by yeast-like fungi remains unclear [2,3,8]. *Candida albicans* remains the most common etiological factor of VVC, but for many years an increasing percentage of NCAC (Non-*Candida albicans Candida*) fungi has been observed among vaginal isolates, such as *Candida glabrata*, *Candida parapsilosis* or *Candida lusitaniae* [5,9,10,11]. Many researchers are inclined to the hypothesis that the ability to form a highly resistant biofilm structure by these strains is one of the causes of therapy failure in VVC [2,3,5,8,9,11,12]. Among virulence factors, the ability to create this structure is one of the most important for their pathogenicity. It seems to be essential primarily in the case of many candidiasis as well as infections related to biomaterials (artificial valves, endoprostheses, intravascular or urinary catheters). What is worth emphasizing is that biofilms are characterized by a high resistance to antifungal drugs, even at very high concentrations [13]. It is noteworthy that the formation of polymicrobial biofilms by *Candida* and bacterial strains is a frequent issue. The most common bacterial strain being isolated from vaginal mixed biofilm is *Lactobacillus* sp. [14]. Hence, research into new antifungal therapies also focuses on anti-biofilm activity.

One of the promising classes of compounds is lipopeptides [15,16,17,18]. They consist of a peptide fragment and conjugated lipid residue (s) and can be divided into subclasses. Ultrashort cationic lipopeptides (USCLs) are among the most effective against fungal strains and consist of a peptide with at most seven amino acid residues with a net positive charge owing to the occurrence of basic amino acids such as arginine or lysine. The most common hydrophobic fragment of USCLS is a fatty acid chain. In effect, USCLs are amphiphilic and can easily interact with the negatively charged pathogen membrane. In the case of fungi, lipopeptides interact with negatively charged residues of sialic acid and phosphatidylinositol found in the cell membrane of these microorganisms [19,20]. Their mode of action is based on the permeabilization of the membrane bilayers, which leads to cell death [16,17,18,21]. They can exhibit plenty of biological properties, such as antibacterial, antifungal, antibiofilm, antiadhesive, anticancer, and surface activities [22]. On the other hand, USCLs can be noticeably lytic to erythrocytes and cytotoxic to normal human cells [16,18,21].

One of the well-studied USCL with a proven antimicrobial activity is C_16_-KKKK-NH_2_ (C_16_-palmitic acid) that contains four L-lysine (K-L-lysine) residues [23,24,25,26,27]. Our previous study on this lipopeptide and its analogs revealed that the substitution of L-lysine by L-arginine (R-L-arginine) residue and disulfide-cyclization can result in compounds with improved antimicrobial activity and selectivity between *Candida* strains and human cells [16]. The lipopeptides with cysteine residues (C-L-cysteine) were cyclized through intramolecular disulfide bridge formation [16]. Based on our previous results, four lipopeptides with the most favorable antifungal properties were selected for further study—C_16_-KKKK-NH_2_, C_16_-KRKK-NH_2_, C_16_-CKKKKC-NH_2_, and C_16_-CKRKKC-NH_2_. Undoubtedly, compounds with different modes of action can potentiate each other’s antifungal activity. It was found that a combination of some lipopeptides (e.g., surfactin) with common antifungal drugs (e.g., azoles) can result in a synergistic effect [28,29,30,31,32,33,34,35,36].

In the context of fungal infections, interactions between conventionally used fluconazole and various AMPs (Antimicrobial peptides) are being investigated more and more frequently [30,37,38,39,40,41,42]. The literature includes several studies on the impact of cationic ultrashort lipopeptides on fungi, including, for example, dermatophytes, but to the best of our knowledge none of these reports concerned isolates from VVC [43].

The aim of the study was to determine the antifungal activity of two linear lipopeptides: C_16_-KKKK-NH_2_ (**L1**), C_16_-KRKK-NH_2_ (**L2**) and their two cyclic analogs: C_16_-CKKKKC-NH_2_ (**C1**) and C_16_-CKRKKC-NH_2_ (**C2**) against 62 clinical strains of various species of *Candida* isolated from Vulvovaginal candidiasis, both in planktonic and biofilm form. Moreover, studies on the potential synergistic or additive effects of combinations of fluconazole with these compounds have been carried out.

## 2. Materials and Methods

### 2.1. Chemicals and Reagents

Chemicals and reagents used in lipopeptides synthesis were: polystyrene resin and amino acids purchased from Orpegen Peptide Chemicals GmbH, Heidelberg, Germany; piperidine, *N*,*N*′-diisopropylcarbodiimide (DIC), OxymaPure, triisopropylsilane (TIS) from Iris Biotech GmbH, Marktredwitz, Germany; *N*,*N*-dimethylformamide (DMF) and diethyl ether from POCH, Avantor, Gliwice, Poland; dichloromethane (DCM) and acetic acid from Chempur, Piekary Slaskie, Poland; hexadecanoic acid (C16) and 1,2-ethanedithiol (EDT) from Merck, Darmstadt, Germany; and trifluoroacetic acid (TFA) from Apollo Scientific, Denton, UK.

### 2.2. Lipopeptides Synthesis

The compounds were obtained by using the method reported previously by Neubauer et al. [16]. Lipopeptides were synthesized manually by solid-phase Fmoc/tBu methodology. Polystyrene resin modified by Rink Amide linker was used as the solid support (loading ca. 1.0 mmol/g). Deprotection of the Fmoc group was performed with a 20% (*v*/*v*) piperidine solution in DMF for 15 min. Acylation was conducted with a mixture of DIC:OxymaPure:Fmoc-AA-OH (mole ratio 1:1:1) dissolved in DMF:DCM (1:1, *v*/*v*) in fourfold excess based on the resin for 1.5 h. Fmoc-L-Arg (Pbf)-OH, Fmoc-L-Lys (Boc)-OH, Fmoc-L-Cys (Trt)-OH, and hexadecanoic acid were used in coupling reactions. After deprotection and coupling reactions, the resin was rinsed with DMF and DCM and subsequently the chloranil test was carried out. The peptides were cleaved from the resin using one of the mixtures: (A) TFA, EDT, TIS and deionized water (92.5:2.5:2.5:2.5, *v*/*v*/*v*/*v*); or (B) TFA, TIS, and deionized water (95:2.5:2.5, *v*/*v*/*v*). Mixture A was used with peptides containing a cysteine residue, whereas mixture B was used for the remaining peptides. Cleavage was accomplished within 1.5 h under stirring. Then the peptides were precipitated with cooled diethyl ether and lyophilized. The crude peptide with cysteine was dissolved in 20% (*v*/*v*) acetic acid solution (0.5 g/L) and oxidized with iodine to obtain the peptide with intramolecular disulfide bridge. The peptides were purified by RP-HPLC. Pure fractions (>95%, HPLC) were collected and lyophilized. The identity of all compounds was confirmed by mass spectrometry (ESI–MS). The sequences of the synthesized lipopeptides were as follows: linear C_16_-KKKK-NH_2_ (L1) and C_16_-KRKK-NH_2_ (L2), cyclic: C_16_-CKKKKC-NH_2_ (C1) and C_16_-CKRKKC-NH_2_ (C2).

### 2.3. Candida Strains

Microbiological assays were performed on 62 clinical isolates of various *Candida* species. All strains were originally isolated from the vaginas of women with Vulvovaginal candidiasis and were deposited in the Internal Collection of the Department of Microbiology, Wroclaw Medical University. Two reference strains of *C. albicans* ATCC 90028 and *C. glabrata* ATCC 15126 (PCM, Polish Academy of Sciences, Wroclaw) were included in all experiments. The vast majority of strains were identified as *C. albicans* (52), while the remaining strains belonged to the NCAC group: i.e., *C. glabrata* (5), *C. lusitaniae* (2), *C. kefyr* (2), and *C. parapsilosis* (1).

The study protocol was approved by the local Bioethics Committee of Wrocław Medical University (No. 774/2018, approval date: 27 December 2018). All experiments were performed in accordance with relevant guidelines and regulations.

### 2.4. Minimum Inhibitory Concentration

Minimum inhibitory concentrations (MICs) of fluconazole and four lipopeptides against *Candida* strains were determined. The research was carried out in accordance with Clinical and Laboratory Standards Institute guidelines [44]. Suspensions of *Candida* strains (subcultured for 24 h on Sabouraud Dextrose Agar with chloramphenicol at a concentration of 100 mg/L) in sterile 0.9% NaCl were diluted in RPMI 1640 (Merck, KGaA, Darmstadt, Germany) to a final concentration of 1–5 × 10^3^ CFU per mL. The test compounds dissolved in DMSO (Merck, KGaA, Darmstadt, Germany) for fluconazole and in sterile distilled water for lipopeptides were diluted in RPMI 1640 on 96-well polystyrene plates to a final range of concentrations 0.125–128 µg/mL (fluconazole) and 0.5–256 µg/mL (USCLs). After the addition of inoculums, all plates were incubated for 24 h at 37 ℃. In the case of fungistatic fluconazole the MIC value is defined as the concentration that inhibits at least 50% of fungal growth. In order to determine the most accurate MIC end-point value, cell densities were determined spectrophotometrically at 530 nm (BiochromAsys UVM 340 Microplate Spectrophotometer, Biochrom Ltd., Holliston, MA, USA). To calculate the MIC value, the following equation was used: (OD_well_-OD_background_)/(OD_K+_-OD_K−_) × 100%, where OD_well_ is the absorbance of the well being assessed, OD_K−_ is the value for the negative control (background), and OD_K+_ is the value obtained in the control positive (strain growth control). Minimum inhibitory concentrations of lipopeptides were the lowest concentrations at which inhibition of fungal growth was noticeable. All experiments were conducted in triplicate.

### 2.5. Minimum Biofilm Eradication Concentration

The determination of minimum biofilm eradication concentrations (MBECs) of all five compounds was performed on 96-well polystyrene flat-bottom plates. Twenty-four-hour cultures of *Candida* were diluted with RPMI 1640 to obtain a final concentration of 1–5 × 10^6^ cells per mL and 100 µL of cell suspension was added into each well of the test plate. The plates were incubated for 24 h at 37 ℃ in order to form a mature biofilm. After incubation, the biofilms were rinsed three times with sterile 0.9% NaCl. Subsequently, fluconazole was added with a final range of concentrations of 1–512 µg/mL, while the lipopeptides concentration ranged between 0.5 and 256 µg/mL. The plates were again incubated overnight at 37 ℃. Visualization of results was carried out with a MTT solution (3-(4,5-dimethyl-2-thiazolyl)-2,5-diphenyl-2H-tetrazolium bromide), Merck, KGaA, Darmstadt, Germany), which is reduced by metabolically active sessile cells of biofilm to purple/navy blue formazan compounds [45]. Yellow MTT solution was added to each well of the plates and incubated for 3 h in the dark at 37 °C. MBECs were the lowest concentrations of the compounds at which no color change was observed (no metabolically active yeast cells were present) as compared to the positive and negative controls. All experiments were conducted in triplicate.

### 2.6. Fractional Inhibitory Concentration Index

The checkerboard method was used to determine fractional inhibitory concentration index (FICi) [46]. Each lipopeptide in combination with fluconazole were prepared on a 96-well polystyrene plate with concentrations serially diluted from 2× MIC to 1/64 MIC for every strain. In each well of the plates prepared as above, different lipopeptide-fluconazole concentrations were obtained. Inoculums of *Candida* strains were prepared as described for the determination of MIC (final concentration of 1–5 × 10^3^ CFU per·mL in RPMI 1640). After the yeast suspension was added, the plates were incubated for 24 h at 37 ℃. Inhibition of *Candida* growth was assessed visually. To calculate the FIC index, the following formula was used:

(1) $$\frac{A}{\mathrm{MIC}\;of\;A}+\frac{B}{\mathrm{MIC}\;of\;B}={\mathrm{FIC}}_{A}+{\mathrm{FIC}}_{B}=\;\mathrm{FIC},$$

(2) $$\mathrm{FIC}\;\mathrm{index}\;=\frac{\sum \mathrm{FIC}}{n}$$

*MIC* values of compound *A* (lipopeptide) and *B* (fluconazole) were obtained in the first part of the research. *A* and *B* values were concentrations of the compounds determined using the checkerboard method. The sum of the ratios of these values (FIC_A_, FIC_B_) was FIC and after it was divided by *n* (number of FICs), the FIC index was obtained. Interpretation of the results was consistent with EUCAST guidelines [47] as follows: FICi ≤ 0.5 indicates synergy (SYN), >0.5 to ≤1.0-addition (ADD), >1.0 to ≤2.0 indifference (IND), and FICi > 2.0 means antagonism (ANT).

## 3. Results

### 3.1. Minimum Inhibitory Concentration

**Figure 1:**
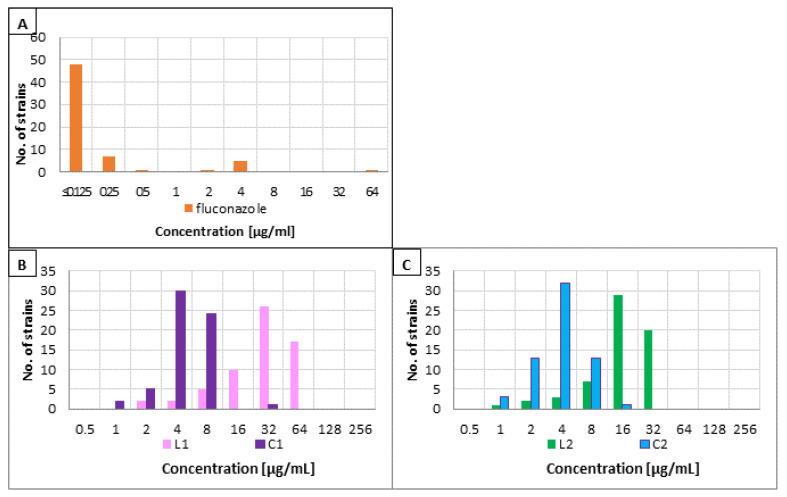
MIC value distribution of the tested compounds: (**A**) fluconazole, (**B**) lipopeptides L1 and C1, and (**C**) lipopeptides L2 and C2.

Fluconazole and four tested lipopeptides exhibited antimicrobial activity against planktonic cultures of all *Candida* strains (Figure 1).

The most common MIC of fluconazole was ≤ 0.125 µg/mL and was determined for 77% of the strains (48/62). All of them were *C. albicans* and only single strains of this species demonstrated a slightly higher MIC, not exceeding 2 µg/mL. One isolate (*C. lusitaniae*) was resistant to fluconazole (MIC = 64 µg/mL). The remaining NCAC strains exhibited minimum inhibitory concentrations of tested azole of 4 µg/mL (all five *C. glabrata* isolates) or less. The distribution of MIC values of fluconazole is presented below in Figure 1A.

Minimum inhibitory concentrations of lipopeptide L1 were in the range 2–64 µg/mL, with 32 µg/mL as the most common value (26/62, ≈42%). No significant differences among various *Candida* species were observed. The cyclic analog of this lipopeptide (C1) exhibited MIC distribution in the lower concentration range between 1 and 32 µg/mL. For almost half (30/62, ≈48%) of the tested strains, MIC of C1 was 4 µg/mL and for 26 isolates (≈42%) it was twice as high 8 µg/mL. No deviations in MICs were observed between different *Candida* species. MIC distribution for L1 and C1 is presented below in Figure 1B.

The second pair of lipopeptides, the one with arginine residue, exhibited less pronounced differences in MIC distribution. MIC concentration range for L2 was 1–32 µg/mL, while for C2 it was 1–16 µg/mL. For nearly 50% (29/62, ≈47%) of strains, minimum inhibitory concentrations of linear lipopeptides were 16 µg/mL, followed by 32 µg/mL (20/62, ≈32%). The most frequent concentration of the cyclic compound was 4 µg/mL (32/62, ≈52%). As with the first pair of lipopeptides, no difference in MIC distribution was observed for individual *Candida* species. MIC distribution of L2 and C2 lipopeptides is displayed in Figure 1C.

To evaluate lipopeptides selectivity, selectivity indices (SI) were calculated as the ration of CM_50_ to GM. Previous results of cytotoxicity (IC50) against HaCaT cell line (immortalized human keratinocytes) were used for this calculation [16]. The results are presented in Table 1.

### 3.2. Minimum Biofilm Eradication Concentration

**Figure 2:**
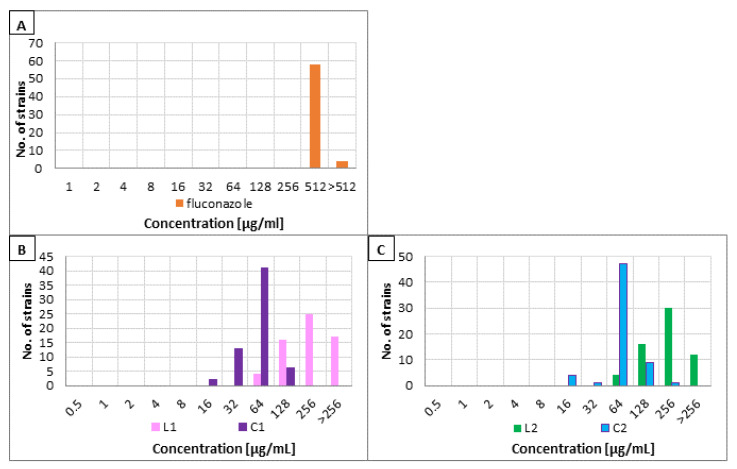
MBEC value distribution of the tested compounds: (**A**) fluconazole, (**B**) lipopeptides L1 and C1, and (**C**) lipopeptides L2 and C2.

Fungistatic fluconazole failed to eradicate the biofilm of *Candida* strains. For almost all strains (58/62, ≈94%), MBEC values were extremely high (512 µg/mL) and for the remaining isolates, eradication concentrations were even higher. The distribution of MBECs is presented below in Figure 2A.

In contrast to fluconazole, all four lipopeptides proved to be effective in biofilm eradication. In the case of the first pair of lipopeptides L1 and C1 (consisting of lysine residues only), similarly to MICs, the obtained MBECs were significantly lower for cyclic lipopeptide than for the linear analog. The most common MBEC value for L1 was 256 µg/mL (25/62, ≈40%), followed by 128 µg/mL (16/62, ≈26%) and concentration above 256 µg/mL (17/62, ≈27%). On the other hand, for the vast majority of strains (41/62, ≈66%), minimum biofilm eradication concentrations of the second lipopeptide (C2) were 64 µg/mL. Again, no differences in MBECs were observed in terms of species. The results of MBEC value determination are shown in Figure 2B.

The MBECs of lipopeptides L2 and C2 were very similar to those described above. In the case of cyclic lipopeptide, MBEC value of 64 µg/mL was definitely dominant (47/62, ≈76%). For the linear parent molecule, the values were more distributed. The most common MBEC was 256 µg/mL (30/62, ≈48%) followed by 128 µg/mL (16/62, ≈26%) and >256 µg/mL (12/62, ≈19%). No differences for *C. albicans* versus NCAC fungi were observed. The results are presented below in Figure 2C.

Geometric means of MBECs were calculated. For MBEC values of USCLs >256 µg/mL, 512 µg/mL was taken into calculations. In the case of fluconazole, 1024 µg/mL value was used respectively for MBECs >512 µg/mL. Importantly, although this mathematical operation includes resistant strains in the calculations, their precise effective concentrations are not known. The calculated GM_MBECs of linear lipopeptides were similar to each other, as well as for the both cyclic analogues. In the case of L1 it was 236.73 µg/mL and 223.86 µg/mL for the L2 lipopeptide. GM_MBEC of lipopeptide with an arginine residue (C2) was 65.45 µg/mL and 56.59 µg/mL for C1.

### 3.3. Fractional Inhibitory Concentration Index (FICi)

**Figure 3:**
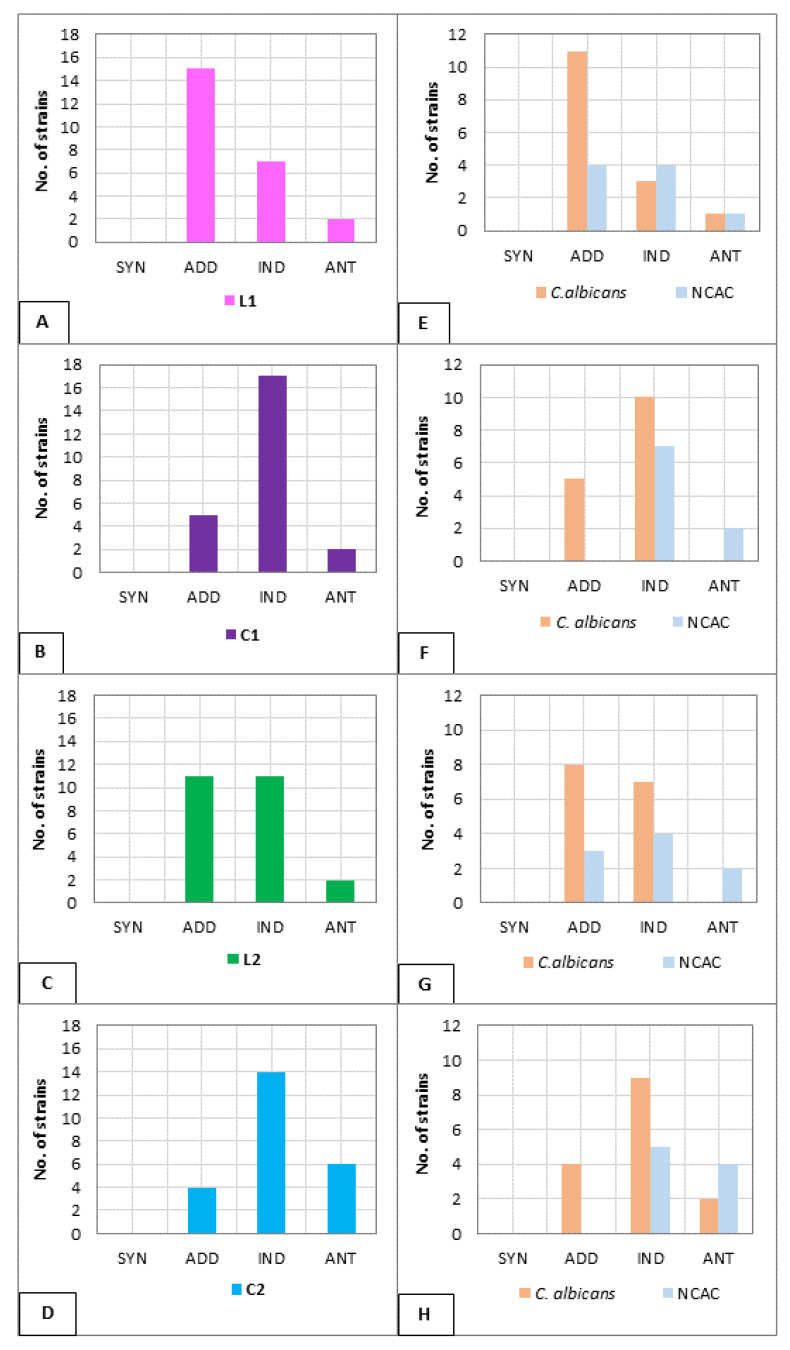
Interpretation of the determined FIC indices (SYN—synergy, ADD—addition, IND—indifference, ANT—antagonism): (**A**) L1; (**B**) C1; (**C**) L2; (**D**) C2; and with differentiation between *C. albicans* and NCAC isolates: (**E**) L1; (**F**) C1; (**G**) L2; (**H**) C2.

To initially asses the interaction of each fluconazole–lipopeptide combination, 24 of the tested strains were randomly selected of which 15 isolates were *C. albicans* and the remaining 9 were from the NCAC group. The interpretation of FIC indices was as follows: FICi ≤ 0.5 indicates synergy (SYN), >0.5 to ≤1.0-addition (ADD), >1.0 to ≤2.0 means indifference (IND), and FICi > 2.0 means antagonism (ANT) [47]. The results are collected in Figure 3.

Although no synergy was observed among the tested fluconazole–lipopeptide pairs against *Candida* strains, an additive effect was determined, especially in the case of *C. albicans* isolates. The distribution of the results on the histograms (Figure 3) clearly indicates that the additive effect is more frequent for both linear lipopeptides combined with fluconazole: L1 (15/24, ≈63%) and L2 (11/24, ≈46%). For cyclic analogs combined with fluconazole, the dominant result was indifference: ≈71% (17/24) for C1 and ≈58% (14/24) for C2. A simultaneous use of cyclic USCLs with fluconazole has an antagonistic or neutral effect against strains of the NCAC group, e.g., *C. glabrata* and *C. kefyr*, in contrast to their linear counterparts. The FIC index determined for *C. albicans* isolates was in agreement with the overall results, with an additive effect obtained mostly for linear lipopeptides.

Based on the obtained results, two lipopeptides were selected for further experiments: linear L1 and its cyclic analog C1. Both USCLs showed more favorable effects (Figure 3, additive effect) against *Candida* in combination with fluconazole than lipopeptides with arginine residue.

**Figure 4:**
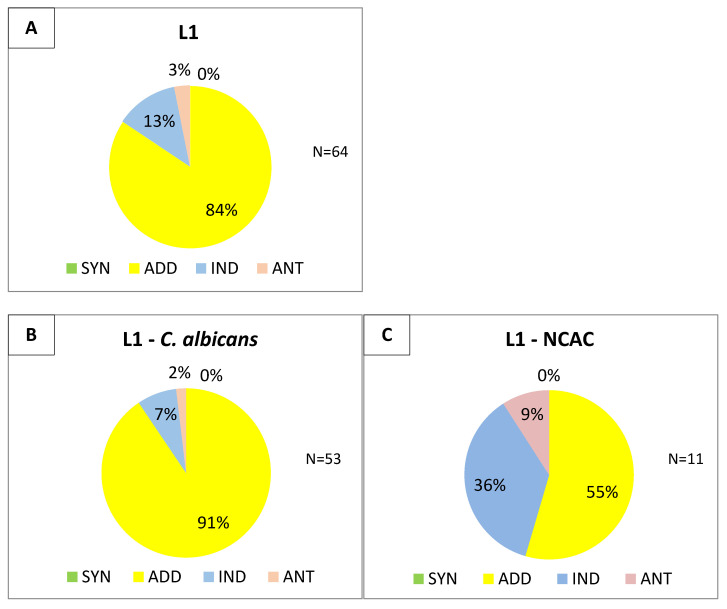
Distribution of FIC indices for combination of fluconazole–L1: (**A**) total—64 strains; (**B**) *C. albicans*—53 strains; (**C**) NCAC—11 strains.

**Figure 5:**
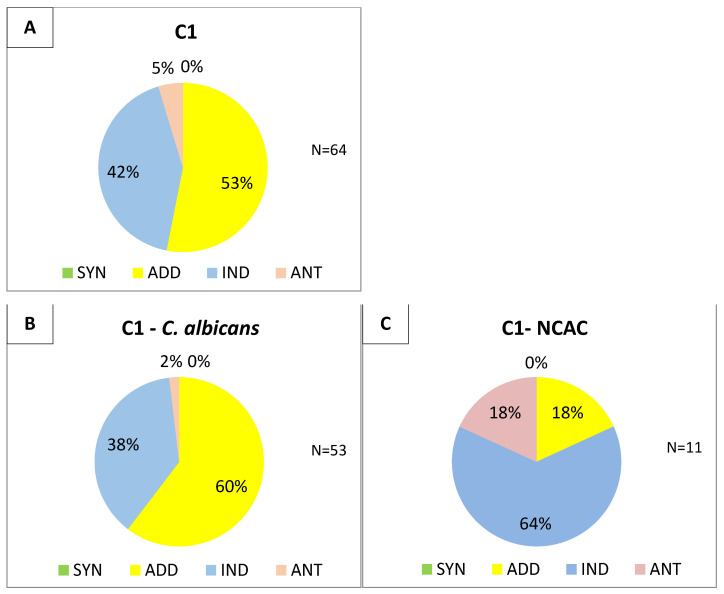
Distribution of FIC indices for the combination of fluconazole–C2; (**A**) total-64 strains; (**B**) *C. albicans*-53 strains; (**C**) NCAC-11 strains.

The FIC indices of combinations of fluconazole–L1 and fluconazole–C1 were determined against the remaining 40 isolates. The results (64 isolates, reference strains included) are shown in Figure 4 and Figure 5.

In the case of the linear lipopeptide, the results obtained for all 64 strains were consistent with those described for 24 isolates (Figure 3). Altogether, an additive effect with fluconazole was dominant (54/64, ≈84%). A similar result was observed for *C. albicans* strains (48/53, ≈91%). Among NCAC fungi, additive effect was the most frequent as well (6/11, ≈55%), but it is difficult to draw strict conclusions due to a relatively small pool of strains. At first, cyclic C2 seemed to have an indifferent effect in combination with the tested azole, but a study on 64 strains revealed that addition is the most common result (34/64, ≈53%) in reference to all *Candida* species and also in the case of *C. albicans* species. Unfortunately, this pair of USCL–fluconazole had still a predominantly neutral effect against NCAC group (7/11, ≈64%).

Interestingly, the FIC index itself is an arithmetic mean of eight obtained individual FIC values. When searching for the most advantageous combination of fluconazole–lipopeptide concentrations, it is the single FIC values (and the corresponding concentrations) that should be taken into account and interpreted. For the vast majority of strains and both USCLs–azole pairs, a synergistic effect could be observed (FIC ≤ 0.5). This applies in particular to the linear lipopeptide L1. An analysis of the most beneficial (the lowest possible) FIC values of fluconazole–L1 showed that synergy between these two compounds is achieved against ≈72% (46/64) of the strains. Consistent results were obtained for *C. albicans* isolates (synergistic effect against 40/53, ≈75% of strains). Synergy was also the most common among NCAC fungi (6/11, ≈55%). In the case of cyclic USCL, synergistic effect occurred less often when single FIC values were analyzed, with the additive effect being dominant. For the fluconazole–C1 combination, synergism was present in ≈23% (15/64) of cases, including in ≈26% (14/53) concerned *C. albicans* and only ≈10% (1/11) NCAC. At the same time, additive effect was achieved in ≈61% (39/64)- for *C. albicans* and in ≈55% (6/11) of the remaining strains. Lipopeptide concentrations corresponding to FIC values indicating synergy were at least four times lower than MICs obtained for these strains. For example, when the MIC value of L1 was 64 µg/mL, a synergistic effect was observed when a combination of 8 µg/mL of lipopeptide and 0.031 µg/mL of fluconazole was used. The concentrations of the discussed fluconazole–L1 pair corresponding to synergistic effect, compared to the obtained MIC values, are shown in Table 2. The corresponding data for the fluconazole–C1 combination were included in the supplementary material as Supplementary Table S1.

## 4. Discussion

Being a local infection not associated with mortality, Vulvovaginal candidiasis (VVC) is a clinical problem that is relatively often underestimated in comparison to other types of infections, including other candidosis [6,10]. With the pathomechanism of VVC still not fully understood and a possibility that biofilm formation could be one of the most crucial virulence factors of *Candida* in the development of this condition, the search for new antimicrobial agents also focuses on anti-biofilm activity [3,5,10,48].

Antimicrobial peptides (AMPs), including lipopeptides, with a broad spectrum of activity and a different mechanism of action compared to traditional antibiotics, are capable of eradication of fungal biofilms [16,17,18,21]. Based on our previous study, ultrashort cationic lipopeptides (USCLs) with the most potent antifungal and antibiofilm activities were selected. Two with linear structure, one modified by replacing one lysine residue (K) with an arginine residue (R)—C_16_-KKKK-NH_2_ (L1) and C_16_-KRKK-NH_2_ (L2), as well as two cyclic analogs—C_16_-CKKKKC-NH_2_ (C1) and C_16_-CKRKKC-NH_2_ (C2) (C-L-cysteine residue) [16].

The literature indicates the possibility of a beneficial effect of combinations of fluconazole with various AMPs against e.g., yeast-like fungi, most likely due to the different mechanisms of action of these two groups of compounds [30,37,38,39,40,41,42]. Meanwhile, to the best of our knowledge, no such experiments have been performed using USCLs against *Candida* isolated from VVC.

With one exception of a single *C. lusitaniae* isolate, all tested strains were found to be susceptible to fluconazole (Figure 1A). This is not an unusual situation; more surprising is the fact of such frequent clinical therapeutic failures with this mycostatic. Considering the multifactorial pathomechanism of vaginal invasion by yeast-like fungi, their ability to form a highly resistant biofilm structure may be the major cause of the ineffectiveness of conventionally used azoles [11,49,50,51]. All four analyzed ultrashort lipopeptides showed activity against *Candida* strains. In the case of cyclic analogs, the achieved concentrations inhibiting the growth of planktonic cells are 2–3 times lower than those of their linear counterparts (Figure 1B,C). On the other hand, when comparing the activity of USCLs consisting of only lysine residues with lipopeptides with one arginine residue, the differences still seem to depend on the cyclic/linear structure of the compared compounds. In the case of cyclic analogs, the differences between MICs for C1 and C2 are virtually unnoticeable, while the antimicrobial activity of linear L2 exceeds that of a lipopeptide consisting exclusively of lysine (L1). This finding is consistent with those of our earlier study in which activity against different *Candida* reference strains was analyzed [16]. Similarly to our previous reports, disulfide-cyclized lipopeptides were substantially more active against both biofilm and planktonic cultures of yeast-like fungi than the corresponding parent molecules. There is an unconfirmed hypothesis that disulfide cyclized USCLs are transported inside the fungal cell, causing degradation of the cell membrane and its interior and leading to cell death [52]. Also, the potential advantage of linear analogs with the arginine residue, observed both by Neubauer and in this study, may support the first reports of the accumulation of protamine (salmon) rich in cationic arginine as necessary for anti-*Candida* activity [52]. The determined SIs (Table 1) of cyclic lipopeptides (5.50 and 8.83) are much higher than those of their linear counterparts (0.28 and 0.89). Those results are in agreement with the literature. It has been shown that linear short cationic lipopeptides with hexadecanoic acid chain exhibited no selectivity between pathogens and normal human cells [16,18,21,53]. It is worth mentioning that for similar USCL, consisting of only two lysine residues (C_16_-KK-NH_2_), antifungal activity was already demonstrated, e.g., against *Cryptococcus neoformans* and dermatophytes [43,54].

Similar conclusions are provided by the analysis of the obtained concentrations of biofilm eradication. While fluconazole failed to deal with *Candida* biofilm (Figure 2A), all lipopeptides tested were capable of eradicating this structure (Figure 2B,C). Again, cyclic analogs exhibited more enhanced antibiofilm activity than linear parent molecules. However, there are no substantial differences between the values of eradicating concentrations obtained for analogs composed only of lysine residues versus compounds enriched with an arginine residue (Figure 1B,C). The calculated GM_MBECs support this thesis. The results of research on biofilm are also consistent with our previous reports [16]. Higher minimum concentrations of compounds obtained for the biofilm structure compared to planktonic cells are not surprising. Biofilms, both bacterial and fungal, are characterized by a much higher resistance to antimicrobial agents than planktonic cells and can be associated with therapeutic failure [9,51].

The present results showed that USCLs in combination with fluconazole can give various effects. The FIC indices obtained in the checkerboard method indicate that an additive antifungal effect was obtained more frequently for a combination of fluconazole and linear lipopeptides than for cyclic ones. Preliminary experiments on 24 random *Candida* strains and combinations of all four USCLs with the tested azole showed that the indifferent antifungal effect against vaginally isolated fungi was predominantly observed with cyclic USCLs (Figure 3B,D)). In the case of the L2 lipopeptide, the additive effect with fluconazole occurred more often than for its cyclic analog (≈46% vs. ≈17%). Moreover, an additive effect was the most frequent with L1 (≈63%). Analyzing the above data with regard to the *Candida* species (15 isolates of *C. albicans* vs. 9 NCAC), the obtained results for cyclic lipopeptides are very similar, both *C. albicans* and other species represented an indifferent effect, while for strains from the NCAC group no additive effect was observed. A comparison of the effect of linear USCLs against *C. albicans* indicates the advantage of the analog consisting of four lysine residues, for which a vast majority has an additive effect (73%). Studies on the linear compound enriched with arginine and combined with fluconazole revealed that it had an additive and indifferent effect on a similar percentage of strains (53% and 47%, respectively). Due to the small number of isolates from the NCAC group, a detailed analysis of the distribution of the obtained results seems unreliable.

Lipopeptide L1 was selected for further studies with fluconazole owing to promising results in the preliminary results discussed above. The cyclic analog was included in this study to learn how different structures of USCLs in combination with fluconazole can affect antifungal activity against strains derived from VVC. Although in the case of both tested cyclic compounds the indifference was definitely the dominant effect, in the case of C1, a negative (antagonistic) effect was observed less frequently than for C2 (8% and 25%, respectively). Hence, testing of the remaining pool of 40 strains was performed on a pair of USCLs composed only of lysine residues. The overall FIC index analysis for the entire pool of 62 isolates and 2 reference strains confirmed the predominant additive effect of the fluconazole–L1 combination: 84% in total, 91% including *C. albicans* strains, and 55% of NCAC, although in this case, the relatively small number of isolates (11) should still be kept in mind. Interestingly, similar results were obtained for the cyclic lipopeptide, for which an additive effect was observed in 53% of cases, of which 60% were against *C. albicans*. In the NCAC group, indifference remained the most frequent result (64%). The literature has described the possibility of a favorable antifungal effect due to combinations of fluconazole with various AMPs [37,38,39,40,41,42]. The use of compounds with different mechanisms of action is generally recommended. Combined antifungal therapy has many potential benefits, such as enhancement of the fungicidal effect and broadening the spectrum of activity, which enable to fight polymicrobial infections, reduce the dose of the compounds, and thus also reduce dose-dependent toxicity as well as overcome the resistance of microorganisms [55,56]. It is noted that antimicrobial peptides interacting with the membrane bilayers could, in a way, sensitize *Candida* cells to fluconazole by increasing azole penetration into the cell, where its molecular target—14α-lanosterol demethylase (Erg enzyme)—is located and involved in ergosterol synthesis. In effect, the composition of the cell membrane changes; it liquefies and increases the permeability for K^+^ and ATP, causing a fungistatic effect [57]. On the other hand, the interaction of fluconazole with the cell membrane may enhance its permeabilization by various AMPs, including lipopeptides, and enhance their fungicidal activity [55,58,59]. Other mechanisms that may be responsible for the synergistic effect of combining triazoles with compounds with a different mechanism of action include sequential inhibition of different stages in the mutual biochemical pathway or simultaneous interaction with the *Candida* cell wall and/or membrane [55]. However, this hypothesis remains unconfirmed as of today.

Last but not least, there is one more interesting aspect of research using the checkerboard method. Knowing the general nature of the interaction of the combination of fluconazole with the lipopeptide against *Candida*, the next step is to select the most favorable and effective concentrations of both compounds to combat fungi. For this purpose, the individual FIC (as the FIC index is the arithmetic mean of eight different FIC values) obtained for a given pair of compounds should be interpreted and the corresponding concentrations selected. In this way, the most favorable (the lowest) FIC for each of the isolates was analyzed in both fluconazole–L1 and fluconazole–C1 combinations. In 72% of strains (75% of *C. albicans*, and 55% of NCAC), there was such a combination of concentrations of linear lipopeptide and FLC for which FIC indicated a synergistic effect (FIC ≤ 0.5). The most beneficial FICs for the cyclic lipopeptide still showed a predominant additive effect (61%), although a synergistic effect was also observed (23%). A detailed analysis revealed that the concentration of L1 in combination with fluconazole that results in synergy is up to four-fold lower (2 vs. 16 µg/mL) than when lipopeptide is used separately (MIC value, Table 2). The literature contains an increasing body of reports about the results of similar studies of interactions of different compounds, not always having any activity against *Candida* alone, indicating a synergistic effect of their combinations with a number of antimycotics, including triazoles. There is a great interest in research on amphiphilic Lactoferrin (LF and its derivatives, cationic peptides), the use of which together with fluconazole (and not only) results in a significant increase in fungistatic activity and a decrease in MIC values [60,61,62,63]. Although the mechanisms responsible for this phenomenon remain unexplained, attention is drawn to the beneficial effects of cationic compounds, such as LF, which may enhance the hydrophobicity of the surface of microbial cells and potentiate the antifungal activity of other compounds [61]. Another example is the advantageous synergistic effect of combinations with fluconazole of such positively charged compounds as microbicidal cationic oligomers, styrylpyridinium compounds, and novel antimicrobial peptides such as KABT-AMP derivatives or ToAP2 [55,59,64,65]. Recently, the synergism of fluconazole with surfactin (SU) against *C. albicans* has been described in more detail. Suchodolski et al. showed that SU binds to chitin and β-glucan on the surface of fungal cells, exposing it to the components of the host’s immune system. However, to achieve the necessary effect, there seems to be required a reduction or complete lack of ergosterol, resulting in the corresponding changes in cell membrane, and this is ensured by the presence of fluconazole [30]. Derivatives of quaternary ammonium compounds (QAC) are other compounds whose activity is similar to that of cationic surfactants and which at the same time have a structure similar to lipopeptides (positive charge, presence of a lipid chain). One representative of this group, compound K21, was recently tested for antifungal activity for the first time. It seems to be an effective alternative to fluconazole against *Candida* strains resistant to this mycostatic. K21 also shows synergism with triazoles towards NCAC, including *C. dubliniensis* and *C. tropicalis*, but no such effect was observed for *C. albicans* [66]. Meanwhile, a combination of another quaternary ammonium compound, domiphen bromide, with miconazole (imidazole) showed a synergistic effect against not only *Candida* planktonic cells, but also a biofilm, although this effect did not occur in the case of triazoles, including fluconazole [67]. On the other hand, the mechanism of action of benzimidazolium-based QAC gemini surfactants was identified as an influence on ergosterol synthesis in a manner similar to that of triazoles. Nevertheless, benzimidazolium-based QACs were more effective and their combination with fluconazole results in a synergistic effect against various *Candida* species [68]. There are also reports in the literature about the synergistic effect of USCL with a structure similar to L1 and L2 with fluconazole and other triazoles, as well as with amphotericin B or terbinafine against *Cryptococcus neoformans* fungi and various representatives of dermatophytes [43,54]. Recently, there have also been reports of the possible synergistic effect between a CEO (citronella essential oil) and ZnO NPs (films based on chitosan with zinc oxide, ZnO, andnanoparticles, NPs) [69]. It is also postulated that the combination of dietary flavonoid, quercetin, with fluconazole is effective against *C. albicans*, including the biofilm created by these strains on the vaginal mucosa in murine Vulvovaginal candidiasis model [70]. However, the mechanism of interaction of the above-mentioned compounds alone and in combination against fungi remains unclear. The literature and our results together clearly demonstrate the enormous potential of ultrashort cationic lipopeptides as compounds enhancing the activity of the existing antimycotics.

Therefore, the results achieved in this work constitute another important premise in the search for antifungal compounds and their combinations with conventional mycobiotics. Moreover, our findings can contribute to the broadening of knowledge in the search for mechanisms involved in the interactions of various cationic compounds with target cells and other antimicrobial compounds. Importantly, the most serious problems to be solved before the actual use of USCLs in the treatment of fungal infections are their relatively high toxicity and unsatisfactory selectivity between microorganisms and human cells [18,21]. The use of combination therapy has a potential to significantly reduce the concentration of lipopeptides effectively against *Candida* and to reduce toxicity towards human cells.

## 5. Conclusions

The results of this study performed with clinical isolates of *Candida* species confirmed high antifungal potency of USCLs, which have previously been studied only with reference yeast strains. Among the four tested lipopeptides, the cyclic compounds C1 and C2 (C_16_-CKKKKC-NH_2_ and C_16_-CKRKKC-NH_2_) showed higher activity against planktonic cells and biofilm of *Candida* isolated from VVC than their linear analogs, L1 and L2 (C_16_-KKKK-NH_2_ and C_16_-KRKK-NH_2_). Both cyclic counterparts were also more selective to pathogens over human cells, as demonstrated by SIs. Although the linear lipopeptide with a single arginine residue appeared to be more active against planktonic cells than the USCL consisting of only four lysine residues, no similar relationship was observed for biofilm-eradicating concentrations. With regard to VVC, it would be undoubtedly worthwhile to conduct studies on the toxicity of these compounds towards vaginal epithelium cell lines and to take into account a larger number of strains from the NCAC group. The study on the interactions of fluconazole combined with lipopeptides showed the advantage of linear USCLs over cyclic ones, especially C_16_-KKKK-NH_2_. The concentrations of the linear lipopeptides causing a synergistic effect against *Candida* species turned out to be at least four-fold lower than when lipopeptides were used separately. Presumably, it would be possible to use a combination therapy, achieving beneficial fungicidal effects owing to the use of compounds with different mechanisms of action, against which the development of resistance would be significantly impeded, at low non-toxic and therefore safer concentrations.

## Figures and Tables

**Table 1:** Geometric mean of MICs (GM_MIC), IC_50_, and selectivity indices (SI) of four tested lipopeptides.

Lipopeptide	GM_MIC [µg/mL]	IC_50_ [16]	SI
L1	26.46	23.5 ± 1.3	0.89
C1	4.89	26.9 ± 1.9	5.50
L2	15.47	4.3 ± 0.9	0.28
C2	3.83	33.8 ± 3.1	8.83

**Table 2:** Concentrations of fluconazole–L1 combination exhibited a synergistic effect against 46 isolates of *Candida* strains.

	FIC		
MIC of L1 [µg/mL]	L1 [µg/mL]	Fluconazole [µg/mL] (Random Order)	No. of Strains Against Which This Combination was Effective
16	2	0.002 or 0.5	2 × *C. albicans* 1 × *C. glabrata*
16	4	0.002 or 0.031 or 0.5	5 × *C. albicans*
32	8	0.002 or 0.031	8 × *C. albicans*
32	4	0.002 or 0.004 or 0.5	6 × *C. albicans* 1 × *C. glabrata*
32	2	0.002 or 0.063 or 1	5 × *C. albicans* 3 × *C. glabrata*
64	16	0.002 or 0.016	3 × *C. albicans*
64	8	0.002 or 0.031	6 × *C. albicans*
64	4	0.002 or 0.004	4 × *C. albicans*
64	2	0.004 or 1	1 × *C. albicans* 1 × *C. lusitaniae*

## Data Availability

The datasets generated during and/or analyzed during the current study are available from the corresponding author on reasonable request.

## Supplementary Materials

The following are available online at https://www.mdpi.com/article/10.3390/pharmaceutics13101589/s1, Table S1. Concentrations of fluconazole–C1 combination exhibited a synergistic effect against 15 isolates of *Candida* strains.
