# Supplementary material for: Antifungal Activity of Linear and Disulfide-Cyclized Ultrashort Cationic Lipopeptides Alone and in Combination with Fluconazole against Vulvovaginal Candida spp
Source: Pharmaceutics. 2021 Sep 30;13(10):1589. doi: 10.3390/pharmaceutics13101589 (PMC8537571; doi:10.3390/pharmaceutics13101589)
Supplement: Supplementary file 1 [file pharmaceutics-13-01589-s001.zip › pharmaceutics-1365878-supplementary.pdf]

pharmaceutics

Supplementary Materials: Antifungal Activity of Linear and Disulfide-Cyclized Ultrashort Cationic Lipopeptides Alone and in Combination with Fluconazole Against Vulvovaginal *Candida* spp.

Paulina Czechowicz, Damian Neubauer, Joanna Nowicka, Wojciech Kamysz, Grażyna Gościniak

Table S1. Concentrations of fluconazole-**C1** combination exhibited a synergistic effect against 15 isolates of *Candida* strains.

	**FIC**		
**MIC of C1** [μg/mL]	**C1** [μg/mL]	**Fluconazole** [μg/mL] (random order)	**No. of Strains Against Which This Combination was Effective**
4	1	0.03125 or 1	1 × *C. albicans*<br>1 × *C. lusitaniae*
8	1	0.001953	2 × *C. albicans*
8	2	0.001953 or 0.003906 or 0.015625 or 0.03125	10 × *C. albicans*
32	4	0.001953	1 × *C. albicans*
